# A Successful Pilot Experiment of Salt Reduction in Tunisian Bread: 35% Gradual Decrease of Salt Content without Detection by Consumers

**DOI:** 10.3390/ijerph18041590

**Published:** 2021-02-08

**Authors:** Jalila El Ati, Radhouene Doggui, Myriam El Ati-Hellal

**Affiliations:** 1 INNTA (National Institute of Nutrition and Food Technology), SURVEN (Nutrition Surveillance and Epidemiology in Tunisia) Research Laboratory, 11 Rue Jebel Lakhdar, Bab Saadoun, Tunis 1007, Tunisia; jalila.elati@yahoo.fr (J.E.A.); doggui.radhouene@gmail.com (R.D.); 2 IPEST (Preparatory Institute for Scientific and Technical Studies), Laboratory Materials Molecules and Applications, P.B. 51, Tunis 2070, Tunisia

**Keywords:** salt reduction, pilot experiment, saltiness perception, Bizerte city, Tunisia

## Abstract

As bread is the most consumed food by the Tunisian population and the major source of salt, a pilot experiment of salt reduction in bread was begun in Bizerte city. Salt analysis in bread collected from Bizerte city was done with the Volhard titration method. A one-way Anova test was carried out to assess salt content changes over time. Application of the salt reduction programme allowed a gradual decrease of salt content in bread by 35% during three years without detection by Tunisian consumers. The salt concentration in bread was then reduced from 1.7 ± 0.2 g/100 g to 1.1 ± 0.1 g/100 g (*p* < 0.0001). The establishment of an effective salt reduction strategy with lifestyle education is needed to reduce hypertension, which is the primary cause of death in Tunisia.

## 1. Introduction

Salt has been extensively proven to be one of the most important causal factors of hypertension [1,2,3]. Several countries have already reduced salt intake [4,5,6,7]. In the Tunisian diet, bread is a staple food and was identified as the most important source of sodium intake. The national food consumption data of 2015 suggested that the daily French bread consumption per person was about 197 g (222 g in urban area and 135 g in rural area). The consumption of all types of bread was 245 g (240 g and 253 g, respectively, in urban and rural areas). French bread intake contributes to 30–50% of the added salt intake (7.1 g/d at the national level) [8]. The Tunisian government has embarked on a major programme to improve the nutritional quality of the Tunisian diet and to combat obesity as well as noncommunicable diseases by reducing the fat, sugar and salt content of the diet [9]. Several actions are being tested in Bizerte city (with about 165,000 inhabitants) as a pilot phase of this national plan, with the reduction of the salt content in bread as being one of these actions.

## 2. Methods and Materials

### 2.1. Bread Collection and Salt Analysis

**Figure 1:**
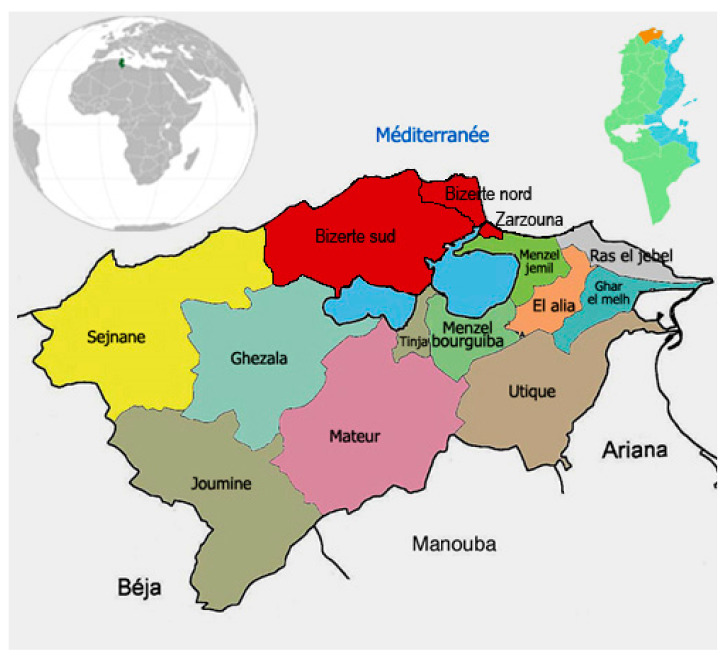
Map of Bizerte city.

The salt reduction initiative was planned for a period of three years (from February 2015 to January 2018) and aimed to reduce the average sodium/salt content in the bread-making process by approximately 30% during the first three months (10% reduction per month) and to maintain reduction for the subsequent 33 months. Twenty-two bakeries from Bizerte city (Figure 1), a northeast area with about 165,000 inhabitants, volunteered to take part in this experiment. French bread was selected for the salt reduction programme as it is the most consumed food (197 g per day per person), contributing to 30–50% of the added salt intake [8]. Bread samples were collected randomly from voluntary bakeries once a week during the first three months and once every three months during the next period of the protocol. Levels of sodium chloride in bread were determined using the Volhard titration method [10]. Briefly, 2.5 g of dried and milled bread was mixed with 29 mL of 0.5 mol L^−1^ silver nitrate and boiled in 15 mL of concentrated nitric acid and 2 mL of potassium permanganate solution. Excess of silver ions was titrated with 0.5 mol L^−1^ ammonium thiocyanate in the presence of diethyl ether and ferric ammonium sulfate indicator. Each sample analysis was carried in triplicate. Reproducibility was estimated through the calculation of the coefficient of variation for the carried sample analysis (CV% = 4.5). Accuracy was evaluated by calculating the mean percentage recovery of bread samples spiked with known amounts of sodium chloride at the beginning and the end of each day’s work. The method showed good percentage recoveries ranging from 95.2 to 108.1.

### 2.2. Saltiness Perception by Tunisian Consumer

During the first three months of the salt reduction programme, overall, 184 consumers (99 women, 85 men) of mean age 37 years agreed to evaluate the saltiness of the salt-reduced bread when they went to purchase from the selected bakeries. The participants were not informed about the salt reduction in bread. From the 184 consumers, 51 persons tested the 10% salt reduction level in the bread-making process during the first week of the pilot action, 39 tested the 20% reduction level in the fifth week, 57 tested the 30% reduction level in the ninth week and 37 tested the 40% reduction level in the thirteenth week. Bread samples were selected randomly from the bread offered for sale in the bakery and cut into 10 mm slices and served on plates. Each participant tasted only one bread sample at a time. The first and the last cut of the loaves were not considered in the saltiness test. Each consumer was asked to fill in a questionnaire, which addressed a single question “Let us know your perception of bread saltiness” with four possible responses (not at all salty, slightly salty, normal-salty and very salty) after rinsing their mouth with tap water.

### 2.3. Data Management and Statistical Analysis

Data management was performed using Stata software version 13.0 (Stata Corporation, College Station, TX, USA, 2013) software. Results are shown as mean ± standard deviation for continuous variables and as percentages for categorical variables. A one-way Anova test was carried out to assess the salt content changes in bread over time.

## 3. Results

**Figure 2:**
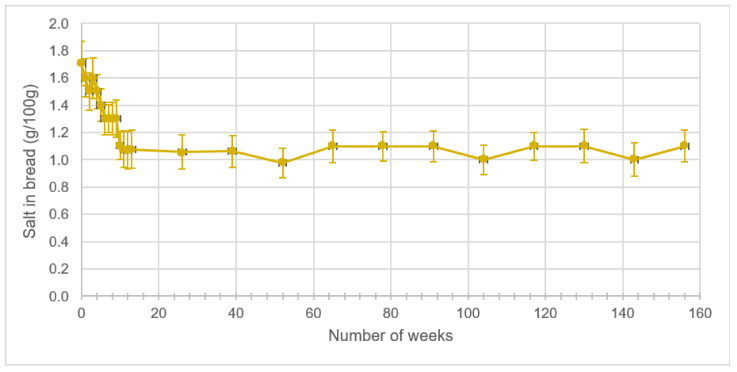
Mean levels and standard deviations of salt in bread collected from Bizerte city after application of the salt reduction programme.

Data on the salt content analysis in bread after application of the salt reduction programme are presented in Figure 2. Before the application of the programme, the mean salt content in bread was 1.7 ± 0.2 g/100 g. Results showed a decrease in the amount of added salt reaching 35% after three months of weekly control (*p* < 0.0001). A stabilization of salt concentration in bread was then noticed with a final salt concentration of 1.1 ± 0.1 g/100 g after three years of subscription to the salt reduction programme.

**Figure 3:**
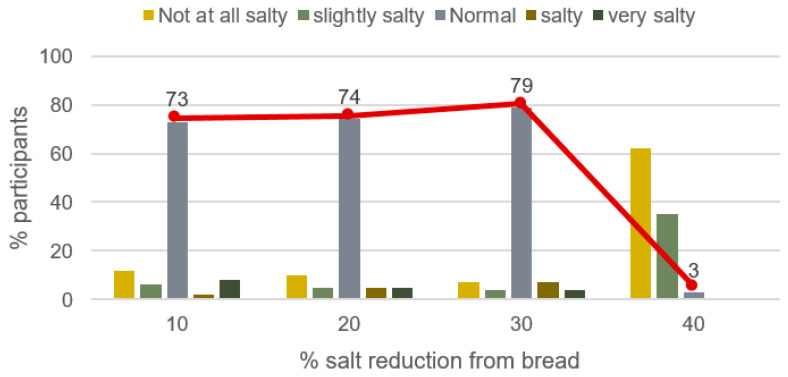
Saltiness perception of salt reduced bread by Tunisian consumers.

Results on bread saltiness perception by Tunisian consumers revealed that salt reduction in the bread-making process was not detected up to the 30% level as 79% of the selected participants found that this salt reduced bread had normal saltiness (Figure 3). However, only 3% of Tunisian consumers who tested the 40% salt reduction level in bread-making process estimated its salinity as normal.

## 4. Discussion

Application of the salt reduction programme in Bizerte city led to an average reduction of added salt in bread of 35% after three years of subscription to this national programme. A final salt concentration of 1.1 ± 0.1% was then achieved. These results are very encouraging when compared to other salt reduction experiments carried out in the world. In Argentina, the average salt content in bread was 2% and French bread accounted for 25% of the total salt intake [11]. A reduction in salt concentration of French bread from 2% to 1.4% was tested and well accepted in the studied population. In Europe, a salt reduction strategy started in Finland in the late 1970s. It involved mass media campaigns, cooperation with the food industry and implementation of salt-labeling legislation [12]. The launch of this legislation has resulted in a 20% reduction in the average salt content in bread from 1.5% to 1.2%. The United Kingdom strategy for salt reduction is considered a model for other countries. It started in 2003 and was based on a set of voluntary targets promoting the gradual reduction in the salt content of processed foods [13]. In the case of white bread, average concentrations of salt decreased from 1.22 ± 0.18% to 1.00 ± 0.10% between 2001 and 2011, which corresponds to a total reduction of 18% in salt level. Following the successful experiments carried out in Finland and the United Kingdom, several other European countries developed their own guidelines by a gradual and sustained reduction of the salt added in processed food. Moreover, the World Health Organization (WHO) has implemented national salt reduction strategies in countries at all income levels to achieve the target of 30% reduction in salt intake by 2025 [14]. Despite the large and increasing number of countries with salt programmes in place (75 until 2015), additional support is required in eastern Mediterranean and African regions where only three countries (Kuwait, Morocco and South-Africa) have assessed salt reduction strategies [15].

In this study, the salt concentration in bread from Bizerte city was gradually reduced by 35% in a period of three months and maintained at this level of reduction during two years and nine months. This rate could be considered satisfactory as the saltiness of bread was considered normal by Tunisian consumers until a 30% salt reduction level (when prepared by bakers). However, a 40% reduction in salt content of bread was detected by almost all participants. Accordingly, the selected bakeries opted for a final level of salt reduction of 30% in the bread-making process during the pilot study.

Salt has several important functions in bread. It controls the rate of fermentation, improves the texture of the product and creates a specific flavor and taste profile [16]. According to Bolhuis et al. (2011), a gradual reduction of salt in bread by up to 52% did not lead to lower consumption of bread over a period of two weeks [17]. Another meta-analysis study conducted on salt-reduced products suggested that reducing the salt level in breads by approximately 40% did not impact consumer acceptability significantly [18]. Pasqualone et al. (2019) tested the effect of salt reduction on quality and acceptability of durum wheat bread. They found that it would be possible to decrease the salt amount of the durum wheat bread formulation from 2 to 1.5% without significantly affecting consumer appreciation [19]. Reduction of salt in bread could be achieved stepwise within a few months or years to be undetected by the consumers, or in a short time by using suitable salt replacers and taste enhancers such as potassium chloride [16]. The use of encapsulated salt is another technological approach to reduce the sodium concentration in bread by creating taste contrast while maintaining saltiness intensity [20]. Despite the limited amounts of reduced salt over a long period of time, the stepwise reduction of added salt in bread remains the simplest strategy which has brought tangible results around the world [15,16].

## 5. Conclusions

A gradual salt content reduction of 35% was achieved in bread collected from 22 voluntary bakeries (out of the 42 bakeries of Bizerte city), without detection by Tunisian consumers. After this encouraging outcome, the challenge is to ensure that this applies to bakeries across all regions of the country. This measure is now under study to be rolled out to all the 24 governorates with the support of Tunisian Union of Industry, Trade and Handicrafts, and the National Federation of Bakers.

## Data Availability

The data presented in this study are available on request from the corresponding author. The data are not publicly available as these data will still be used for the generalization of the experiment.
